# Follow-up of Secundum ASD, Muscular VSD, or PDA Diagnosed During Newborn Hospitalization

**DOI:** 10.21203/rs.3.rs-3871102/v1

**Published:** 2024-01-18

**Authors:** Jacob Faultersack, Christine Johnstad, Xiao Zhang, Margaret Greco, john hokanson

**Affiliations:** University of North Carolina at Chapel Hill; Intermountain Healthcare; University of Wisconsin–Madison; University of Wisconsin–Madison; University of Wisconsin–Madison

**Keywords:** Echocardiography, Newborn, Secundum Atrial Septal Defect, Muscular Ventricular Septal Defect, Patent Ductus Arteriosus

## Abstract

**Background:**

The ideal follow-up of neonates who have a secundum atrial septal defect (ASD), muscular ventricular septal defect (VSD) or patent ductus arteriosus (PDA) remains uncertain.

**Methods:**

Newborns with findings limited to a secundum ASD, muscular VSD and/or PDA on their last birth hospital echocardiogram and at least one outpatient follow-up echocardiogram performed between 9-1-17 and 9-1-21 were evaluated and patient follow-up assessed through 9-1-23.

**Results:**

95 babies met inclusion criteria. 43 babies had a secundum ASD, 41 had a muscular VSD and 54 had a PDA at newborn hospital discharge. 39/95 had more than one intracardiac shunt. 56 were discharged from care, 26 were still in follow-up and 13 were lost to recommended follow-up.

No patients required intervention during the follow-up period of 2 to 6 years. Of those 43 with a secundum ASD 16 (37.2%) had demonstrated closure of the ASD and 13 (30.2%) were discharged from care with and ASD < 3.5 mm in diameter. 3/43 infants with secundum ASD had a defect large enough to easily warrant further follow-up.

**Conclusion:**

Even in this group who had early clinical follow-up recommended by a pediatric cardiologist, no baby discharged from their birth hospitalization with a secundum ASD, muscular VSD or PDA needed any intervention from 2 to 6 years of follow-up. Ongoing follow-up with echocardiography of those infants with a secundum ASD is of greater value than of those with muscular VSD or PDA.

## Background

Echocardiography is an essential tool in the diagnosis of congenital heart disease in newborns and is vital to early diagnosis of critical congenital heart diseases which require surgical or catheter intervention within 28 days of birth (1). However, the majority of infants in the newborn nursery who receive an echocardiogram do not require early intervention and the number of infants undergoing echocardiography has risen steadily (2), necessitating a discussion on the management of non-critical findings on echocardiography of the newborn.

The cost of echocardiograms can be significant, particularly for those who are uninsured or underinsured. If the findings on neonatal echocardiography can be better classified into those where follow-up imaging is or is not needed, there is potential for savings in health care costs for families and health care facilities. Additionally, continued evaluation and follow up of hemodynamically insignificant echocardiographic findings may put children at risk for vulnerable child syndrome (3) (4, 5). Setting a framework for infants receiving an initial echocardiogram that provides clinicians with reliable guidelines for when further evaluation is appropriate has the potential to decrease unnecessary testing while allowing clinicians to be comfortable referring for further evaluation in those cases where it is appropriate.

A survey conducted in 2020 found that many pediatric cardiologists recommend follow up echocardiography in newborns with small intracardiac shunts even if long term cardiac issues were unlikely (6). The recommended follow-up interval was highly variable ranging from two weeks to three years for secundum atrial septal defects (ASD), two weeks to one year for small muscular ventricular septal defects (VSD) and one week to one year for small patent ductus arteriosus PDA. These follow-up visits typically included echocardiography (6). These three entities are common and when small rarely require intervention (7–14). The available literature on the topic does not address the scenario in which more than one intracardiac shunt is present.

In the past, there was limited guidance for the follow up of neonatal echocardiographic findings that are unlikely to be hemodynamically significant. The American College of Cardiology (ACC) recently released clinical practice guidelines for secundum ASDs (15) VSDs (16) and PDAs (17) that provide clinicians with frameworks for clinical follow-up. These guidelines suggest follow-up at one year without testing if asymptomatic for babies with secundum ASD (15), discharge from care for trivial “silent” PDA, follow-up every 3–6 months without echocardiography for small PDA (17) and include the option not to have scheduled cardiology follow-up for patients with small muscular VSDs(16). In this study, we review the outcomes of babies managed prior to the publication of these clinical practice guidelines.

## Methods

We reviewed the echocardiograms of those babies born at Unity Point Meriter Hospital (Madison, Wisconsin, USA) from 9/1/2017 to 9/1/2021 who had a newborn echocardiographic diagnosis limited to secundum ASD, muscular VSD and/or PDA on their last birth hospital echocardiogram and had at least one subsequent outpatient pediatric cardiology clinic with echocardiography at American Family Children’s Hospital (Madison, WI, USA) from 9/1/17 to 9/1/21. Cardiology follow-up appointments based on newborn echocardiography were made prior to newborn hospital discharge and included echocardiography. Patients seen in the outpatient cardiology clinic would typically have that echocardiogram performed before being evaluated by the cardiologist.

There was no size limit exclusion for the secundum ASD, muscular VSD or PDA. Patients were excluded if their echocardiographic findings were limited to a patent foramen ovale only. Patients were also excluded if they had any other diagnosis necessitating outpatient cardiology follow-up or required transfer to American Family Children’s Hospital for inpatient care prior to their outpatient visit. There was no exclusion by weight or gestational age.

We performed a retrospective chart review of all birth hospital echo reports, all children’s hospital echocardiogram reports and cardiology clinic notes. All echocardiographic data was reported using the SyngoDynamics reporting system (Siemens Medical Solutions USA, Inc, Princeton NJ, USA). Classification as secundum ASD, muscular VSD, and/or PDA was determined by the last birth hospital echocardiogram report. Outpatient follow-up was assessed through 9/1/23, allowing for at least two years of follow-up after the first outpatient cardiology visit. Based on clinic notes and follow up echocardiogram reports, each patient was categorized as discharged from care, still in follow-up or lost to follow-up. Patients could remain in follow-up at the discretion of the clinic cardiologist even if the initial ASD, VSD or PDA had closed if other concerns arose. Echocardiograms were read by one of twelve pediatric cardiologists who had trained at eight different pediatric cardiology fellowship programs.

For those babies with a diagnosis of a secundum ASD, the maximum diameter of the ASD was measured on the last newborn hospitalization echocardiogram and all subsequent outpatient echocardiograms. The greatest diameter of the ASD on two-dimensional imaging from any subcostal view was recorded.

This study was approved by both the Unity Point Meriter Hospital Institutional Review Board and the University of Wisconsin Health Sciences Minimal Risk Institutional Review Board.

Descriptive statistics were calculated on patients’ demographics and overtime clinical metrics for all babies and by their current status (i.e. discharged from care, in on-going care and lost to follow-up). Chi square test for categorical variables and ANOVA test for continuous variables were performed to compare clinical outcomes by indication of initial echocardiography. All analyses were performed with the statistical software STATA/SE 16.1 (StataCorp, College Station, TX). A p-value of 0.05 was defined as significant.

## Results

There were 19,198 births at Unity Point Meriter from 9-1-17 to 9-1-21. 1091 babies (5.7%) had at least one echocardiogram during their birth hospitalization during that time period. After the inclusion and exclusion criteria were applied, 95 babies remained to form the final study population. Patients in this cohort were generally seen in close outpatient follow-up with a mean age at first outpatient echocardiogram of two months and a maximum age at first outpatient echocardiogram of six months. The study cohort included only those babies with an ASD, muscular VSD or PDA where follow-up was recommended, which is presumably a subset of babies at the highest risk for long term consequences of an ASD, VSD or PDA. The demographics of the study population is shown in Table 1.

The indication for the initial echocardiogram at the birth hospitalization did not have a statistically significant impact on the number of echocardiograms performed or the final clinical disposition of the patient as shown in Supplemental Table 1.

A total of 274 echocardiograms were reviewed (124 in the newborn hospitalization and 150 during outpatient follow-up). 56 patients were discharged from follow-up cardiology evaluation, 26 were still in ongoing follow-up and 13 were lost to the follow-up that was recommended at their first outpatient pediatric cardiology visit (Supplemental Table 2). Patients with secundum ASD were over-represented in the group lost to follow-up with 6/19 (31.6%) of those with a diagnosis limited to a secundum ASD having been lost to scheduled follow-up (Table 2).

Ongoing follow-up was recommended for 39/95 (41.5%). The diagnosis at the final birth hospital echocardiogram did not influence the chance of discharge from follow-up, the mean number of outpatient echocardiograms performed or the age at last outpatient follow-up (Supplemental Table 3).

At birth hospital discharge, 43 babies had a secundum ASD, 41 babies had a muscular VSD, and 54 babies had a PDA. 39/95 babies (41.1%) had more than one intracardiac shunt on their last birth hospital echocardiogram.

No infants required medical, catheter, or surgical intervention in follow-up ranging from 2 to 6 years. Echocardiography was performed at all outpatient cardiology visits with a mean of 1.5 echoes per patient in follow-up and with 38/95 patients (41%) having two or more outpatient echocardiograms. None of the patients still in follow-up have a VSD or PDA that is likely to require intervention. None of the six patients still in follow-up for a PDA had a heart murmur on physical examination at the last pediatric cardiology clinic visit.

Additional attention was directed to the 43 infants diagnosed with a secundum ASD on their last newborn hospitalization echocardiogram (Table 2). 3/43 (7.0%) had a secundum ASD with a maximum diameter greater than 5 mm on their last outpatient echocardiogram (6.2 mm at 26 months, 8.6 mm at 1 month and 11 mm at 3 months). All three had normal physical examinations on their last cardiology visit with a normal second heart sound and no audible murmur. Their maximum ASD diameters on their last newborn hospitalization echocardiograms were 7.7, 8.3 and 10.9 mm respectively.

Of those 43 with a secundum ASD on their last newborn hospitalization echocardiogram, 16 (37.2%) had an outpatient echocardiogram with no evidence of an atrial level shunt. 13 (32.6%) were discharged from care despite a residual atrial shunt (mean maximum diameter 2.6, range 1.1–3.4 mm). 9 (20.9%) were lost to follow-up before resolution of their ASD was demonstrated (mean maximum diameter 3.6 mm, range 1.4–8.6 mm). 4 (9.3%) were in ongoing follow-up (mean maximum diameter 5.4 mm, range 1.7–11 mm).

Of those 22 babies with a secundum ASD diameter of => 6 mm at their last newborn hospitalization echocardiogram, 3 (13.6%) had an ASD diameter of => 6 mm at last follow-up. None of the 21 babies with a secundum ASD diameter of < 6 mm at their last newborn hospitalization echocardiogram had a significant increase in their ASD size, with the maximum diameter reported at 4.1 mm at last follow-up.

## Discussion

Our patient population had early echocardiographic follow-up of secundum ASD, muscular VSD and/or PDA in newborns similar to previous survey reporting (6). This study was limited by the small sample size but was focused on those intracardiac shunts in which spontaneous closure is common but in which ongoing follow-up is frequently recommended and is unique in identifying patients with more than one intracardiac shunt. The presence of multiple intracardiac shunts may have increased the likelihood of echocardiographic follow-up or prompted earlier follow-up, yet none of the patients with more than one shunt required intervention.

As was likely the case in many centers, our cardiologists did not have a uniform approach for when to recommend follow-up of secundum ASD, muscular VSD or PDA or when to discharge from care. Neither the results of the 2020 neonatal echocardiography survey (6) nor the ACC clinical practice algorithms (15–17) were available at the time they made these decisions. Because of the retrospective nature of the study, we could not accurately determine which factors might have influenced their clinical recommendations. As outpatient echocardiographic follow-up was recommended by a clinician for all of these patients, they represent a group perceived to be at higher risk for complications or with a lower chance for spontaneous closure. That 19/95 (20%) babies in this cohort had more than one echocardiogram during their newborn hospitalization supports that increased level of concern. Consequently, the follow-up recommended was often sooner even than that from the 2020 survey (6) and considerably earlier than recommended by the ACC clinical practice algorithms(15–17). Even in what was perceived to be a group in need of close outpatient follow-up, no clinical interventions were necessary.

Although an argument that early follow-up might decrease those lost to follow-up, even in this group who had already had one outpatient echo, 13/95 were lost to further recommended follow-up after their initial outpatient cardiology visit. This was particularly true in the subgroup who had a secundum ASD but neither a muscular VSD nor PDA.

Our assessment of the maximum diameter of the secundum ASD on the two-dimensional subcostal view is a crude assessment of the atrial septal anatomy at best, but not without merit by providing at least a rough size estimated of the defect. Obtaining an accurate diameter with two-dimensional imaging is particularly difficult with the smallest of atrial level shunts which might be considered clinically insignificant.

Of the three intracardiac shunts, a secundum ASD is the one in which ongoing follow-up seems the most appropriate. Of the 43 babies with a diagnosis of a secundum ASD on their last newborn hospital discharge echocardiogram, none required intervention in the follow-up period and three had secundum ASDs on their last outpatient echocardiogram that were large enough to easily justify ongoing follow-up. Unlike the case of a muscular VSD or PDA, a normal physical exam in childhood cannot definitively exclude a hemodynamically significant secundum ASD. In hindsight, many of the outpatient echocardiograms in patients with secundum ASDs were performed well before a decrease in size or spontaneous closure could have been expected based on the existing literature (10, 11). That literature suggests that spontaneous closure of small to moderate secundum ASD often takes several months. This follow-up was also well before the follow-up at one year of age (without planned echocardiography) recommended by the ACC clinical practice algorithm(15). That guidance recommends follow-up echocardiography be deferred to between ages 3 and 5 years of age unless other clinical concerns arise earlier.

41/95 patients had a muscular VSD either in isolation or with a secundum ASD or PDA, but none required intervention. This lack of intervention is consistent with that previously reported(14) suggesting that intervention is typically not needed in small to moderate muscular ventricular septal defects. It also supports the recommendations of the ACC Clinical Practice algorithm for ventricular septal defects(16).

54/95 patients had a PDA at the time of their last birth hospital echocardiogram, and none required intervention in the follow-up period. It should be noted that the population of infants with PDA requiring closure via catheter intervention during their newborn hospitalization was not included in this study as they met exclusion criteria of transfer during birth hospitalization. Although the mean age at the last birth hospital echocardiogram was 6.5 days, many of the patients had their echocardiograms performed prior to the median age of PDA closure reported by Nagasawa et al of 27 hours for boys and 45 hours for girls (18). The follow-up performed in this group was also typically performed well before the 3–6 months interval recommended in the ACC Clinical Practice Algorithm (17). The six patients still in follow-up for a PDA all had a normal physical examination (the so-called silent PDA).

In all intracardiac shunt groups, repeat echocardiography was performed earlier than can be easily supported by the available literature or the recently published ACC clinical practice algorithms. Arguably, this cohort might be at a higher risk for complications as a cardiologist recommended what might now be considered very early follow-up of their intracardiac shunt. Even in this group in which early follow-up was recommended, no intervention was needed during follow-up between 2 and 6 years after the first outpatient echocardiogram, suggesting that a more delayed follow-up would not have altered clinical care.

Although pediatric cardiology follow-up of these newborns without echocardiography was not a typical part of our group’s clinical practice during this study, pediatric cardiology follow-up without echocardiography was recommended by a minority of respondents to the 2020 survey(6). Such an approach would allow for clinical assessment of the patient and identify those rare individuals who would develop symptoms related to an intracardiac shunt and those in which ongoing follow-up would be more easily justified.

## Conclusion

Early echocardiographic follow-up of secundum ASD, muscular VSD and PDA diagnosed in the newborn period as seen in this cohort and as has previously been recommended by pediatric cardiologists (6) involves significant resource utilization that may be of limited benefit. Although ongoing echocardiographic follow-up in patients with secundum ASD at a later age appears to be appropriate, planned, ongoing echocardiographic follow-up of muscular VSD and PDA appears to be of limited value. The clinical outcome of this cohort supports logic behind and the use of the recently published ACC Clinical Practice Guidelines for ASD(15), VSD(16) and PDA(17).

## Figures and Tables

**Table 1.** Demographics and Clinical Information (N=95)

Sex, N (%)	
Male	44 (46.3)
Female	51 (53.7)
Newborn hospital echoes per baby, mean (SD), Range	1.3 (1.0) Range 1–7
Babies with more than one newborn hospital echo, N (%)	19 (20.0)
Gestational age at last newborn hospital echo (weeks), mean (SD), Range	39.0 (1.5), Range 34.6–42.3
Weight on last newborn hospital echo (kilograms), mean (SD), Range	3.1 (0.6), Range 1.1–4.8
Age on last newborn hospital echo (days), mean (SD), Range	6.2 (14.9), Range 0–70
Indication for Newborn hospital echo, N (%)	
Murmur	33 (34.7)
Follow-up after fetal echo	30 (31.6)
Trisomy 21	6 (6.3)
Pulmonary Hypertension	5 (5.3)
Family history	4 (4.2)
Postnatal arrhythmia	3 (3.2)
Failed CCHD screening	2 (2.1)
Prenatal arrhythmia	2 (2.1)
Other	10 (10.5)
Diagnosis at Last Birth Hospital Echocardiogram	
Secundum ASD only	19
Secundum ASD with muscular VSD	7
Secundum ASD with muscular VSD and PDA	4
Secundum ASD with PDA	13
Muscular VSD only	15
Muscular VSD with PDA	15
PDA only	22
Outpatient echoes per baby, mean (SD), Range	1.6 (0.9), Range 1–7
Patients with more than one outpatient echo, N (%)	39 (41.0)
Age at first outpatient echo (days), mean (SD), Range	61.5 (32.6), Range 5–182
Weight at first outpatient echo (kilograms), mean (SD), Range	4.8 (1.2), Range 2.7–8.0
Age at last outpatient echo (days), mean (SD), Range	263.1 (376.6), Range 31–1398
Clinical Status	
Discharged from follow-up	56
Not discharged from follow-up	26
Lost to follow-up	13

**Table 2:** Outcome of Patients with Neonatal Diagnosis of a Secundum Atrial Septal Defect

Shunts Present	ASD Diameter on Last Newborn Echo (mm)	Age at Last Newborn Echo (days)	ASD Diameter on Last Outpatient Echo (mm)	Age Last Outpatient Echo (days)	Status
A	9.8	69	3.1	182	DC
A	8.9	1	4.1	1133	LTFU
A	8.3	1	8.6	34	LTFU
A	8	3	0	455	FU
A	7.7	3	6.1	791	FU
A	7.4	2	1.7	102	FU
A	7.1	1	0	81	DC
A	7	0	3.2	40	DC
A	6.9	6	0	783	FU
A	6.9	44	0	886	DC
A	6.5	5	2.9	105	LTFU
A	6.4	2	2.8	282	DC
A	5.9	2	4.1	74	LTFU
A	5.7	2	1.4	78	LTFU
A	5.1	2	3.4	69	DC
A	5	2	0	1860	DC
A	4.1	3	2.6	41	LTFU
A	3.7	13	2.8	110	DC
A	3.1	3	0	94	DC
A P	10.9	49	11	89	FU
A P	8.4	70	3.5	212	LTFU
A P	6.5	1	0	1100	DC
A P	6.4	0	2.4	90	DC
A P	5.2	3	0	788	DC
A P	4.8	1	2.2	56	DC
A P	4.5	2	0	69	DC
A P	3.9	3	2.4	58	DC
A P	3.8	1	3.4	69	DC
A P	2.9	1	0	120	DC
A P	2	2	2.6	84	LTFU
A P	1.8	1	1.1	32	DC
A V	8.7	4	0	236	LTFU
A V	8	1	2.5	69	LTFU
A V	7.3	2	2.7	32	FU
A V	6	2	2.2	43	DC
A V	5.5	2	0	223	FU
A V	4.2	2	3.1	46	DC
A V	2.2	2	0	93	DC
A V P	9.1	2	0	211	DC
A V P	6.8	1	2.6	46	DC
A V P	4.2	2	0	815	DC
A V P	4	2	1.4	212	DC
A V P	3.6	2	0	175	DC
					
Mean	5.9 mm	7.5 days	2.1 mm	283 days	
Range	1.8 to 10.9 mm	1 to 70 days	0 to 11 mm	32 to 1860 days	

A = Secundum ASD only, AP = Secundum ASD and PDA, AV = Secundum ASD and muscular VSD, AVP = Secundum ASD, muscular VSD and PDA, DC = Discharged from Care, FU = Still in Cardiology Follow-up, LTFU = Lost to Follow-up

## Abbreviations

ASD: Atrial Septal Defect
VSD: Ventricular Septal Defect
PDA: Patent Ductus Arteriosus

## References

[1] Kondo M, Ohishi A, Baba T, Fujita T, Iijima S. Can echocardiographic screening in the early days of life detect critical congenital heart disease among apparently healthy newborns? BMC pediatrics. 2018;18:359. Epub 2018/11/21. 30453920 10.1186/s12887-018-1344-z PMC6241044

[2] Fenster ME, Hokanson JS. Heart murmurs and echocardiography findings in the normal newborn nursery. Congenit Heart Dis. 2018;13:771–5. Epub 2018/07/25. 30039518 10.1111/chd.12651

[3] Coon ER, Quinonez RA, Moyer VA, Schroeder AR. Overdiagnosis: how our compulsion for diagnosis may be harming children. Pediatrics. 2014;134:1013–23. Epub 2014/10/08. 25287462 10.1542/peds.2014-1778

[4] Pearson SR, Boyce WT. Consultation with the specialist: the vulnerable child syndrome. Pediatrics in review / American Academy of Pediatrics. 2004;25:345–9. Epub 2004/10/07. 10.1542/pir.25-10-345 15466135

[5] Bergman AB, Stamm SJ. The morbidity of cardiac nondisease in schoolchildren. N Engl J Med. 1967;276:1008–13. Epub 1967/05/04. 6022469 10.1056/NEJM196705042761804

[6] Hokanson JS, Ring K, Zhang X. A Survey of Pediatric Cardiologists Regarding Non-emergent Echocardiographic Findings in Asymptomatic Newborns. Pediatr Cardiol. 2022;43:837–43. Epub 2022/01/10. 34999921 10.1007/s00246-021-02795-8

[7] Connuck D, Sun JP, Super DM, Kirchner HL, Fradley LG, Harcar-Sevcik RA, Incidence of patent ductus arteriosus and patent foramen ovale in normal infants. Am J Cardiol. 2002;89:244–7. Epub 2002/01/17. 11792356 10.1016/s0002-9149(01)02214-7 PMC10246183

[8] Wang NK, Shen CT, Lin MS. Results of echocardiographic screening in 10,000 newborns. Acta Paediatr Taiwan. 2007;48:7–9. Epub 2007/01/01. 19653409

[9] Hagen PT, Scholz DG, Edwards WD. Incidence and size of patent foramen ovale during the first 10 decades of life: an autopsy study of 965 normal hearts. Mayo Clin Proc. 1984;59:17–20. Epub 1984/01/01. 6694427 10.1016/s0025-6196(12)60336-x

[10] Hanslik A, Pospisil U, Salzer-Muhar U, Greber-Platzer S, Male C. Predictors of spontaneous closure of isolated secundum atrial septal defect in children: a longitudinal study. Pediatrics. 2006;118:1560–5. Epub 2006/10/04. 17015547 10.1542/peds.2005-3037

[11] Radzik D, Davignon A, van Doesburg N, Fournier A, Marchand T, Ducharme G. Predictive factors for spontaneous closure of atrial septal defects diagnosed in the first 3 months of life. J Am Coll Cardiol. 1993;22:851–3. Epub 1993/09/01. 8354823 10.1016/0735-1097(93)90202-c

[12] Roguin N, Du ZD, Barak M, Nasser N, Hershkowitz S, Milgram E. High prevalence of muscular ventricular septal defect in neonates. J Am Coll Cardiol. 1995;26:1545–8. Epub 1995/11/15. 7594083 10.1016/0735-1097(95)00358-4

[13] Zhao QM, Niu C, Liu F, Wu L, Ma XJ, Huang GY. Spontaneous Closure Rates of Ventricular Septal Defects (6,750 Consecutive Neonates). Am J Cardiol. 2019;124:613–7. Epub 2019/06/19. 31208700 10.1016/j.amjcard.2019.05.022

[14] Frandsen EL, House AV, Xiao Y, Danford DA, Kutty S. Subspecialty surveillance of long-term course of small and moderate muscular ventricular septal defect: heterogenous practices, low yield. BMC pediatrics. 2014;14:282. Epub 2014/11/06. 25370708 10.1186/1471-2431-14-282 PMC4289403

[15] Plummer S PA, Sachdeva R, Zaidi A, Statile C Clinical Practice Algorithm For the Follow-up of Unrepaired and Repaired Secundum Atrial Septal Defects. 2022. Epub March 8, 2022.

[16] Birnbaum Brian M, FACC; Hahn Eunice, MD; Sheth Shreya, MD, FACC; Steele Jeremy, MD; Sachdeva Ritu, MBBS, FACC; Parthiban Anitha, MBBS, FACC; Statile Christopher, MD, FACC; Zaidi Ali N., MD, FACC. Clinical Practice Algorithm For the Follow-Up of Unrepaired and Repaired Ventricular Septal Defects. 2023.

[17] Hancock H AA, Massarella D, Moshin S, Parthiban A, Smith C, Statile C, Zaidi A, Sachdeva R. Clinical Practice Algorithm For the Follow-Up of Unrepaired and Repaired Patent Ductus Arteriosus. 2022. Epub October 31, 2022.

[18] Nagasawa H, Hamada C, Wakabayashi M, Nakagawa Y, Nomura S, Kohno Y. Time to spontaneous ductus arteriosus closure in full-term neonates. Open Heart. 2016;3:e000413. Epub 2016/05/31. 27239325 10.1136/openhrt-2016-000413 PMC4874051

